# α7 nAChR mediated Fas demethylation contributes to prenatal nicotine exposure-induced programmed thymocyte apoptosis in mice

**DOI:** 10.18632/oncotarget.21526

**Published:** 2017-10-05

**Authors:** Han-Xiao Liu, Sha Liu, Wen Qu, Hui-Yi Yan, Xiao Wen, Ting Chen, Li-Fang Hou, Jie Ping

**Affiliations:** ^1^ Department of Pharmacology, Wuhan University School of Basic Medical Sciences, Wuhan 430071, China

**Keywords:** prenatal nicotine exposure, α7 nAChR, thymocyte apoptosis, TET2, Fas promoter demethylation

## Abstract

This study aimed to investigate the effects of prenatal nicotine exposure (PNE) on thymocyte apoptosis and postnatal immune impairments *in vivo* and further explore the epigenetic mechanisms of the pro-apoptotic effect of nicotine *in vitro*. The results showed that PNE caused immune impairments in offspring on postnatal day 49, manifested as increased IL-4 production and an increased IgG1/IgG2a ratio in serum. Enhanced apoptosis of total and CD4+SP thymocytes was observed both in fetus and in offspring. Further, by exposing thymocytes to 0–100 μM of nicotine *in vitro* for 48 h, we found that nicotine increased α7 nicotinic acetylcholine receptor (nAChR) expression, activated the Fas apoptotic pathway, and promoted thymocyte apoptosis in concentration-dependent manners. In addition, nicotine could induce Tet methylcytosine dioxygenase (TET) 2 expression and Fas promoter demethylation, which can be abolished by TET2 siRNA transfection. Moreover, the α7 nAChR specific antagonist α-bungarotoxin can abrogate nicotine-induced TET2 increase, and the following Fas demethylation and Fas-mediated apoptosis. In conclusion, our findings showed, for the first time, that α7 nAChR activation could induce TET2-mediated Fas demethylation in thymocytes and results in the upregulation of Fas apoptotic pathway, which provide evidence for elucidating the PNE-induced programmed thymocyte apoptosis.

## INTRODUCTION

Inflammatory and immune diseases have become an important and ever growing health problem over the past decades [1]. Increasing evidence has identified risks for immune diseases are, to a certain extent, determined by the prenatal adverse environment, causing programed functional alteration of the immune system [2–4]. Normal development of thymocytes in fetus is essential for the establishment of postnatal immune function [5]. Prenatal exposure to xenobiotics could interfere with the normal developmental programming of thymocyte [3, 5]. During the development process, the thymocytes can be divided into several subpopulations based on the expression of CD4 and CD8: CD4^−^CD8^−^ double negative cells (DN), CD4^+^CD8^+^ double positive cells (DP) and single positive cells (SP, CD4^+^SP or CD8^+^SP). All of these subpopulations express the Fas receptor, which can induce cell apoptosis through death-inducing signaling complex assembly and later the activation of the caspase-8. Caspase-8 then directly activates other members of the caspase family, among which caspase-3 plays a central role in the final execution-phase of thymocyte apoptosis [6, 7]. It is believed that this Fas/caspase-mediated apoptosis is crucial for regulating thymocyte development [6]. In addition, some reports have suggested that the enhanced apoptosis of fetal thymocytes might persist into postnatal life and, consequently, might cause immune impairments and enhance the risk of immune diseases [5, 8] .

Cigarette smoke can cause many health problems; however, 25–29% of pregnant women insist on smoking and approximately half of pregnant women are exposed to second-hand smoke [9, 10]. Of women who smoked during the last 3 months of pregnancy, 48% reported smoking more than 6 cigarettes per day (data from *Pregnancy Risk Assessment Monitoring System*). Epidemiological investigations have shown that cigarette smoke exposure during pregnancy is a significant threat to fetal thymocyte development [11]. Experimental studies further demonstrated that prenatal cigarette smoke exposure could promote thymocyte apoptosis permanently [12]. Among all smoke products, nicotine is widely accepted as one of the aversive components that perturb fetal development because nicotine can quickly pass the placental barrier to reach the embryo and accumulate in fetal circulation [13, 14]. In addition, nicotine was reported as one of the leading candidates of cigarettes for influencing apoptotic processes of immune cells, and the thymus is one of the toxic target organs of nicotine [15, 16]. However, to date, little is known about whether and how nicotine contributes to the effects of pregnant smoke exposure on fetal thymus apoptosis and postnatal immune function.

Nicotine induces its actions by selectively binding to its receptors known as the nicotinic acetylcholine receptors (nAChRs) [17]. Studies have shown that immune cells express the α7 nAChR. Although no studies have reported that α7 nAChR can induce Fas-mediated apoptosis in thymocytes, considerable evidence has suggested that α7 nAChR could directly induce apoptotic pathways in multiple organs [17]. The α7 nAChR activation could trigger the influx of extracellular Ca2^+^, which is associated with the upregulated Fas expression [18]. In addition, in a prenatal cigarette smoke exposure model, researchers reported that α7 nAChR was activated and the increase of α7 nAChR was consistent with the increased caspase expression and apoptosis ratio [19]. Therefore, we speculated that the α7 nAChR might exert the pro-apoptotic effects of nicotine on thymocytes by upregulating the Fas expression and the Fas apoptotic pathway.

Extensive data from both human and animal studies indicate that a prenatal altered intrauterine environment during the most important phases of fetal organ development can potentially induce permanent changes in gene expression [20]. Firm evidence has shown that these changes in gene expression are mediated through epigenetic mechanisms [21]. It is clear that the Fas expression is activated by DNA demethylation modification in the Fas promoter at the level of transcription [22]. Moreover, perinatal distress has been reported to increase the DNA demethylation modification of Fas and alter Fas expression permanently, resulting in exceeding apoptosis in many physiological processes in the offspring [23]. The results from our previous studies and other labs demonstrated that nicotine is one of the representative modulators of DNA methylation and could induce abnormal methylation modifications in multiple genes [24–27]. However, whether nicotine could disturb the normal DNA methylation level of Fas and consequently contribute to upregulated Fas expression has not yet been reported and warrants further investigation.

In the present study, we investigated the prenatal nicotine exposure (PNE)-induced programmed thymocyte apoptosis in fetuses and female offspring, and analyzed the changes of immune responses following antigen challenge in PNE offspring. To study the direct effects and molecular mechanisms of nicotine on thymocyte apoptosis, thymocytes were treated with nicotine *in vitro*. The upregulation of the Fas apoptotic pathway and the Tet methylcytosine dioxygenase (TET) 2-mediated demethylation of the Fas promoter were observed. Further, by using the α7 nAChR specific antagonist α-bungarotoxin and TET2 siRNA, we reported, for the first time, that α7 nAChR activation could increase Fas expression and the Fas apoptotic pathway through TET2-mediated DNA demethylation in thymocytes. This work will help to characterize the developmental toxicity of nicotine on the fetal thymus and provide evidence for the underlying mechanisms for the developmental origin of immune diseases.

## RESULTS

### In vivo

#### PNE increased the ratio of immunoglobulin (Ig) G1/IgG2 and the concentration of interleukin (IL)-4 in serum

**Figure 1:**
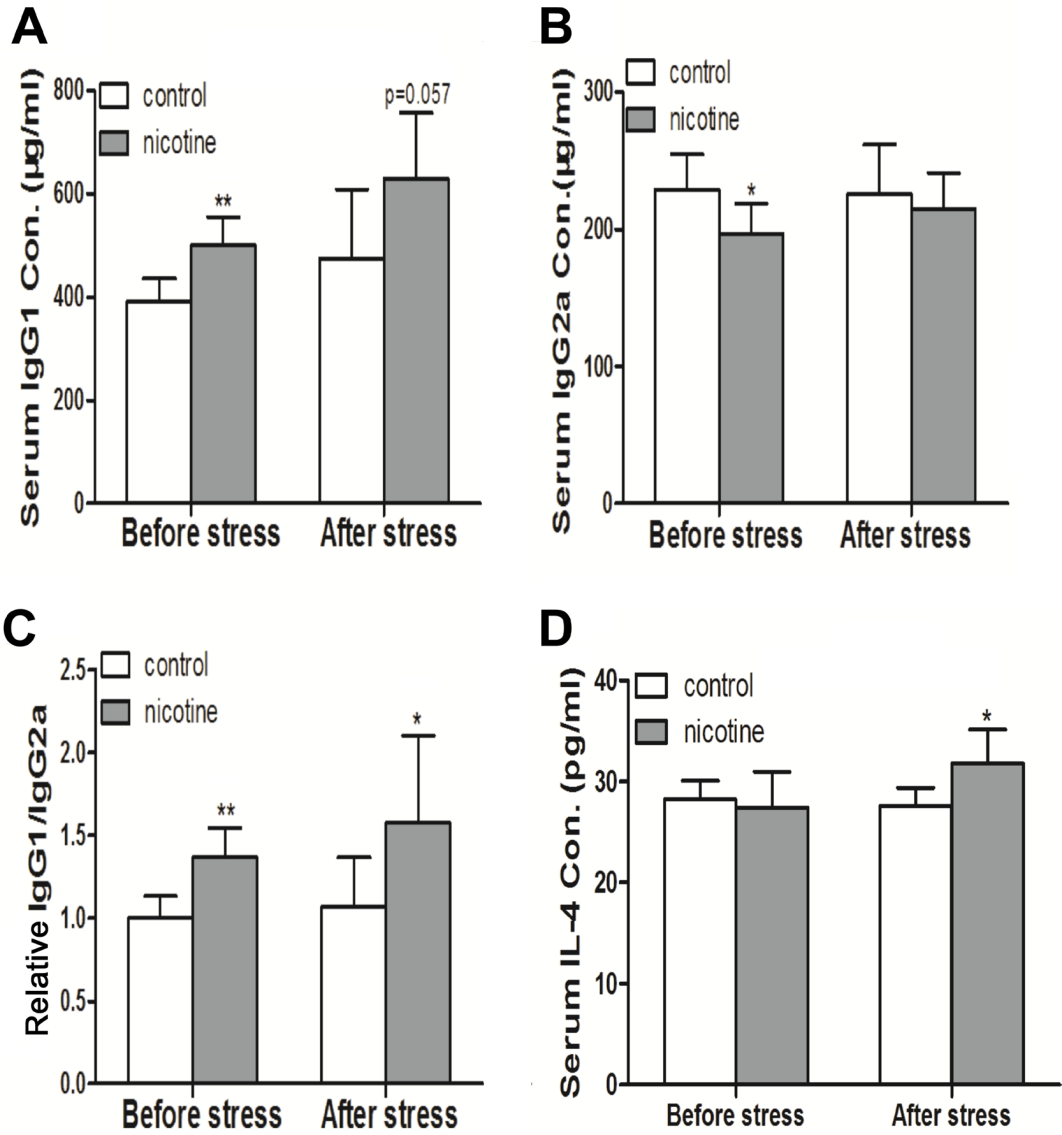
Effects of prenatal nicotine exposure on serum IgG1, IgG2a, and IL-4 production of female offspring on PND 49 The pregnant mice were exposed to 3mg/kg/d nicotine from GD 0 to GD 18. After birth, the female offspring were maintained until PND 49. Half of the female offspring on PND 42 were immunized with *S. pneumoniae* for 7 days and were sacrificed after anesthesia on PND49. The contents of IL-4, IgG1, and IgG2a in serum were then analyzed. Difference among multiple groups was analyzed with one-way ANOVA. (**A**) Serum IgG1 concentration; (**B**) Serum IgG2a concentration; (**C**) The relative IgG1/IgG2a ratio which was standardized by taking the IgG1/IgG2a ratio of the control group as 1; (**D**) Serum IL-4 concentration. Mean ± SD, *n* = 7–8. ^*^P < 0.05, ^**^P < 0.01 vs control.

Prenatal exposure to cigarette smoke could cause abnormal IL-4 and Ig production during inflammation development in offspring [28]. To investigate the inflammatory responses of PNE offspring, we immunized the offspring with Streptococcus pneumoniae vaccine (*S*. *pneumoniae*) on postnatal day (PND) 42 and detected the serum contents of IL-4, IgG1 and IgG2a on PND 49. As shown in Figure 1A and 1B, increased the levels of serum IgG1 before and after immunization and decreased the level of serum IgG2a before immunization were observed in female PNE offspring compared to the control (*P* < 0.01, *P* < 0.05, *P* = 0.057). As a result, all of the PNE offspring (before and after immunization) exhibited a higher ratio of IgG1/IgG2a compared to the control (*P* < 0.01, *P* < 0.05, Figure 1C). After immunization, a significant increase in serum IL-4 concentration was also observed in PNE offspring (*P* < 0.05, Figure 1D).

#### PNE altered the thymocyte phenotypes and increased thymocyte apoptosis in female offspring on PND 49

**Figure 2:**
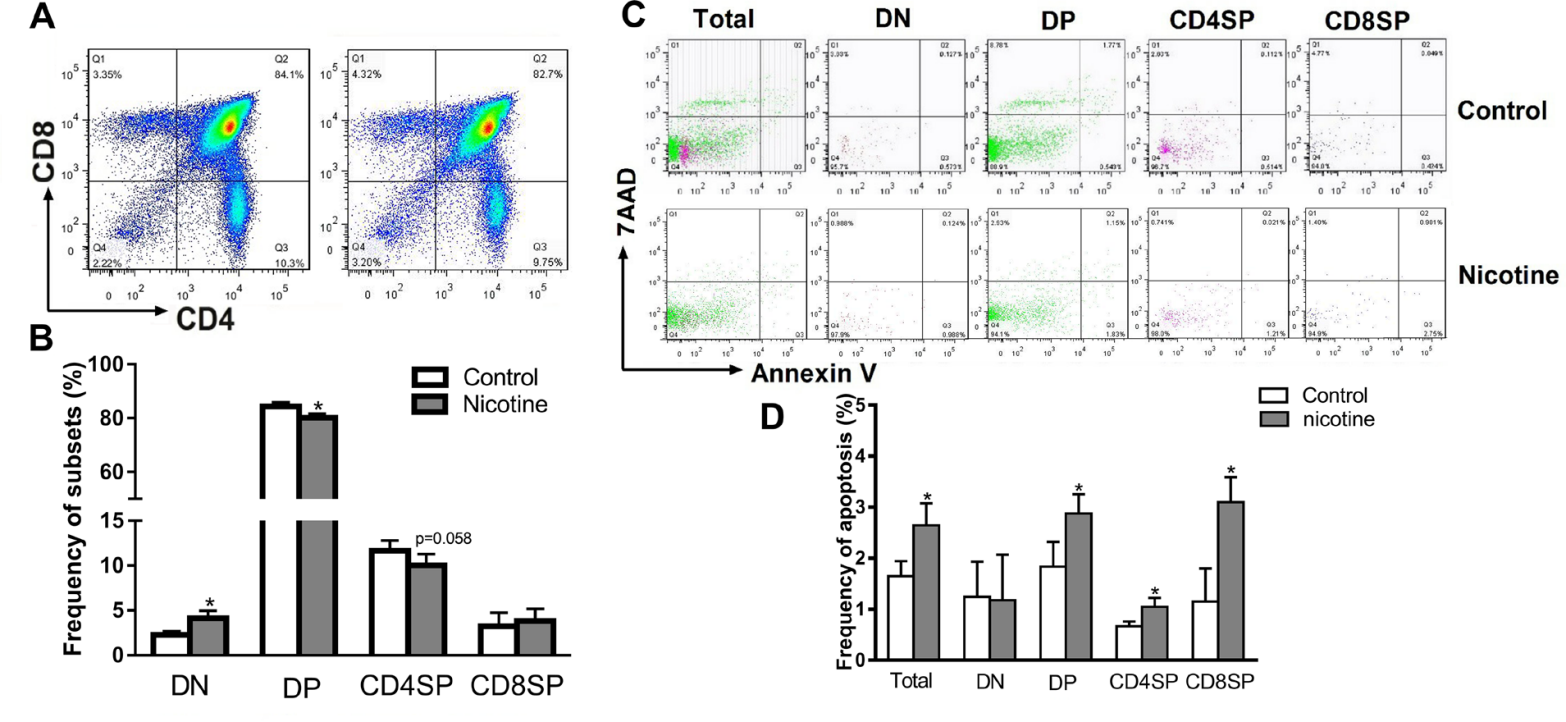
Effects of prenatal nicotine exposure on the thymocyte phenotypes and apoptosis frequency in female offspring on PND 49 Female offspring were sacrificed after anesthesia on PND 49 and thymuses were harvested and dissociated over the 40 μm stainless steel to prepare single thymocyte suspensions. Then the thymocytes were stained with 100 μl of antibody cocktail (FITC-CD3, APC-CD4 and PE-cy7-CD8) to identify thymocyte phenotypes. Annexin V/PE apoptosis detection kit was also used to determined thymocyte apoptosis frequency. (**A**) Typical flow diagram of thymocyte phenotypes; (**B**) Frequency of thymocyte subsets; (**C**) Typical flow diagram of thymocyte apoptosis; (**D**) Apoptosis frequency of thymocytes. The difference was analyzed with *t*-test. Mean ± SD, *n* = 3–4. ^*^P < 0.05 vs control.

Thymocyte apoptosis and thymopoiesis suppression were reported to be responsible for the immune impairments [29]. We analyzed the phenotypes and apoptosis of thymocytes on PND 49 by flow cytometry. As shown in Figure 2A and 2B, the PNE female offspring exhibited a higher percentage of DN (the immature subpopulation of thymocytes) and lower percentages of DP and CD4^+^SP than that of control (*P* < 0.05, *P* = 0.058). Moreover, on PND 49, the apoptosis percentages of total thymocytes, DP, and CD4^+^SP of PNE female offspring were higher than that of control (*P* < 0.05, Figure 2C and 2D).

#### PNE altered the thymocyte phenotypes and increased thymocyte apoptosis in the fetus

**Figure 3:**
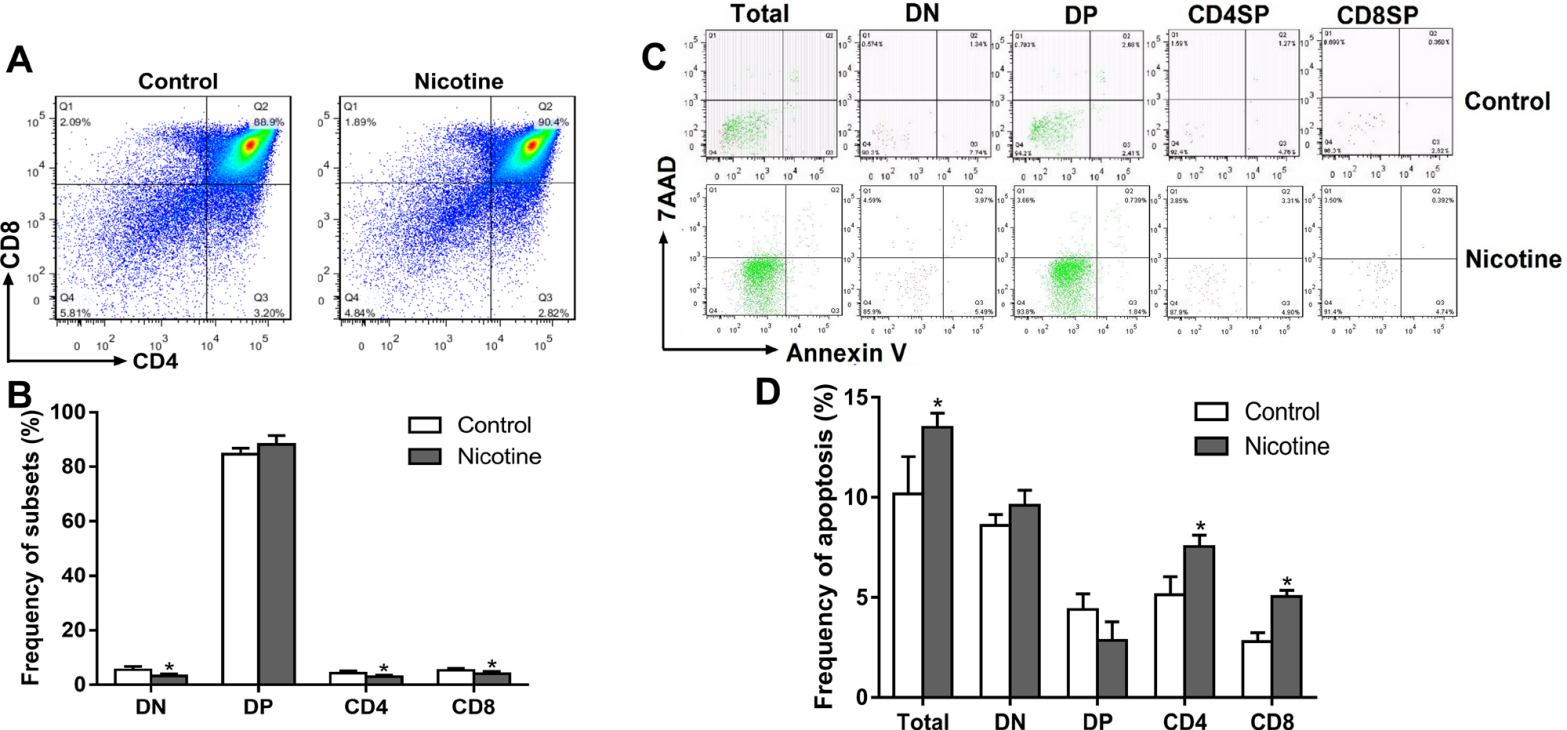
Effects of prenatal nicotine exposure on the thymocyte phenotypes and apoptosis frequency in the fetus on GD 18 Pregnant mice were sacrificed after anesthesia on GD 18. Fetuses were removed quickly from the uterus and decapitated after euthanasia. The fetal thymus from each littermate were collected and pooled into one sample for flow cytometry detection. (**A**) Typical flow diagram of thymocyte phenotypes; (**B**) Frequency of thymocyte subsets; (**C**) Typical flow diagram of thymocyte apoptosis; (**D**) Apoptosis frequency of thymocyte. The difference was analyzed with *t*-test. Mean ± SD, *n* = 3–4. ^*^P < 0.05 vs control.

We further analyzed the thymocyte phenotypes and apoptosis in the fetus on gestational day (GD) 18 to explore the programming effects of PNE on fetal thymus development. As shown in Figure 3A and 3B, PNE fetuses exhibited significantly lower percentages of DN, CD4^+^SP and CD8^+^SP thymocytes (*P* < 0.05). Consistent with the phenotypes, the apoptosis percentages of total thymocytes, CD4^+^SP and CD8^+^SP in PNE fetuses were increased compared with the control (*P* < 0.05, Figure 3C and 3D).

### In vitro

#### Nicotine treatment enhanced thymocyte apoptosis

**Figure 4:**
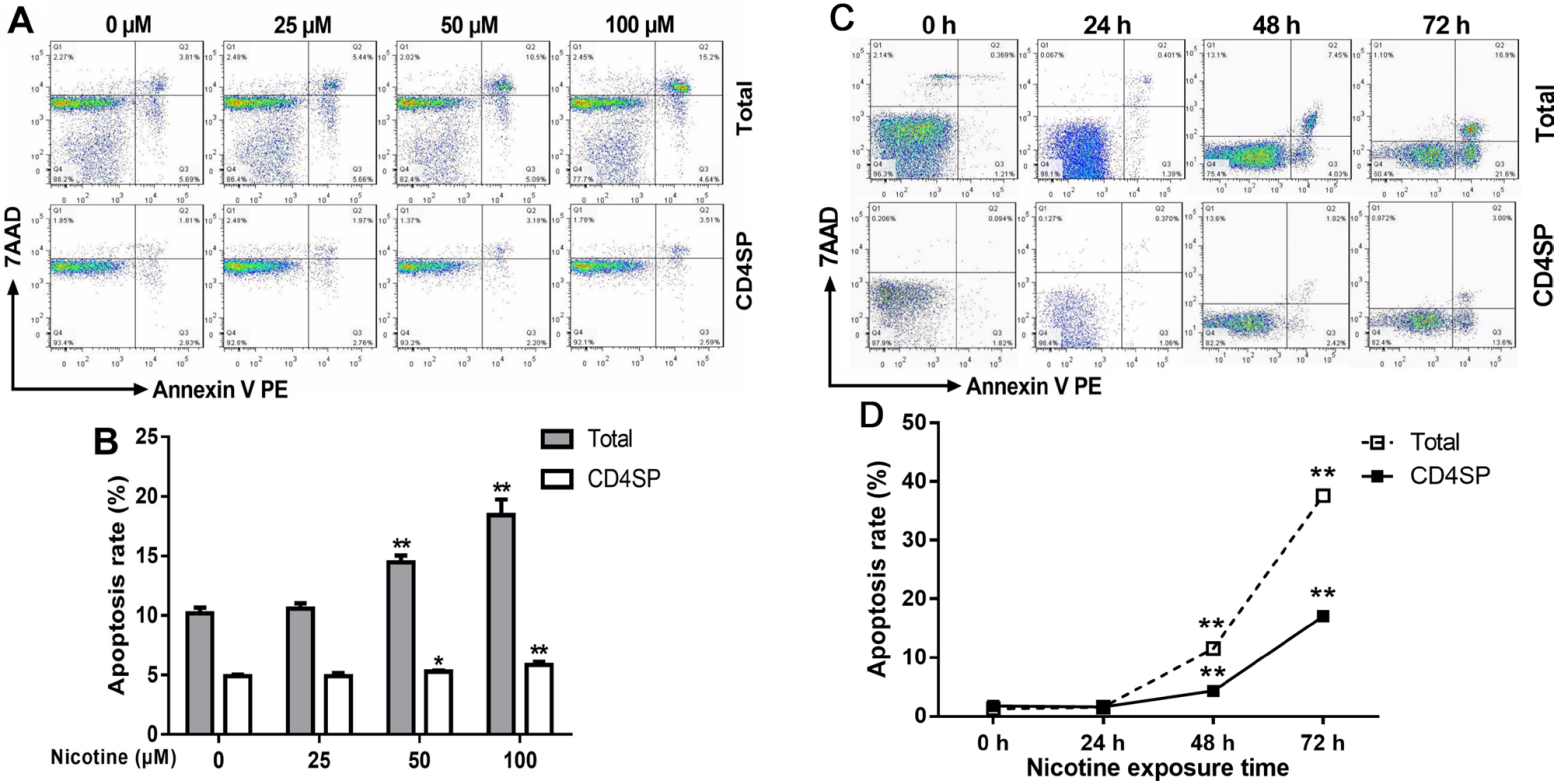
Effect of nicotine treatment *in vitro* on the apoptosis of primary thymocytes Thymus lobes dissected from infant mice at 3 weeks of age were gently dissociated over the stainless steel sieve. The cells were treated with different concentrations (25, 50 and 100 μM) of nicotine for 48 hours or 50 μM of nicotine for 24 h, 48 h and 72 h. The apoptosis frequency was determined by flow cytometry. (**A**, **C**) Typical flow diagram of thymocyte apoptosis; (**B**) Effects of different concentrations of nicotine (0–100 μM) on thymocyte apoptosis; (**D**) Effects of 50 μM nicotine exposure for different time (0, 24, 48, 72 h) on thymocyte apoptosis. The difference was analyzed with *t*-test. Mean ± SD, *n* = 3–4. ^*^P < 0.05, ^**^P < 0.01 vs control.

According to our *in vivo* results, prenatal nicotine exposure could induce programmed apoptosis of total and CD4^+^SP thymocytes. Additionally, nicotine could pass the placental barrier due to its high lipid solubility and act on fetal thymocytes directly [14, 16]. Accordingly, to investigate the direct pro-apoptotic effect of nicotine on thymocytes and the mechanisms, we cultured thymic primary cells from infant mice at 3 weeks of age, and treated the cells with 25–100 μM nicotine for 48 h to analyze the dose-effect of nicotine on thymocyte apoptosis or 50 μM nicotine for 24–72 h to analyze the time-effect of nicotine on thymocyte apoptosis. Thymocyte apoptosis was determined by flow cytometry. The dose-effect results showed that compared to the vehicle (Phosphate-buffered saline, PBS), 50 and 100 μM nicotine significantly increased the apoptosis rates of total thymocytes to 13.9% (*P* < 0.01) and 17.3% (*P* < 0.01), respectively (Figure 4A and 4B). Meanwhile, 50 and 100 μM nicotine also promoted CD4^+^SP thymocyte apoptosis to 5.29% (*P* < 0.01) and 5.85% (*P* < 0.01), respectively. The time-effect results showed that 50 μM nicotine treatment for 24 h had few effects on the apoptosis of thymocytes, while 48 and 72 h treatment of 50 μM nicotine clearly augmented total thymocyte apoptosis to 11.6% (*P* < 0.01) and 31.7% (*P* < 0.01) respectively and CD4^+^SP thymocyte apoptosis to 4.31% (*P* < 0.01) and 17.1% (*P* < 0.01) respectively, compared with the control (Figure 4C and 4D). These results illustrated that nicotine promoted apoptosis in both concentration-dependent and time-dependent manners.

#### Nicotine treatment upregulated the Fas apoptotic pathway

**Figure 5:**
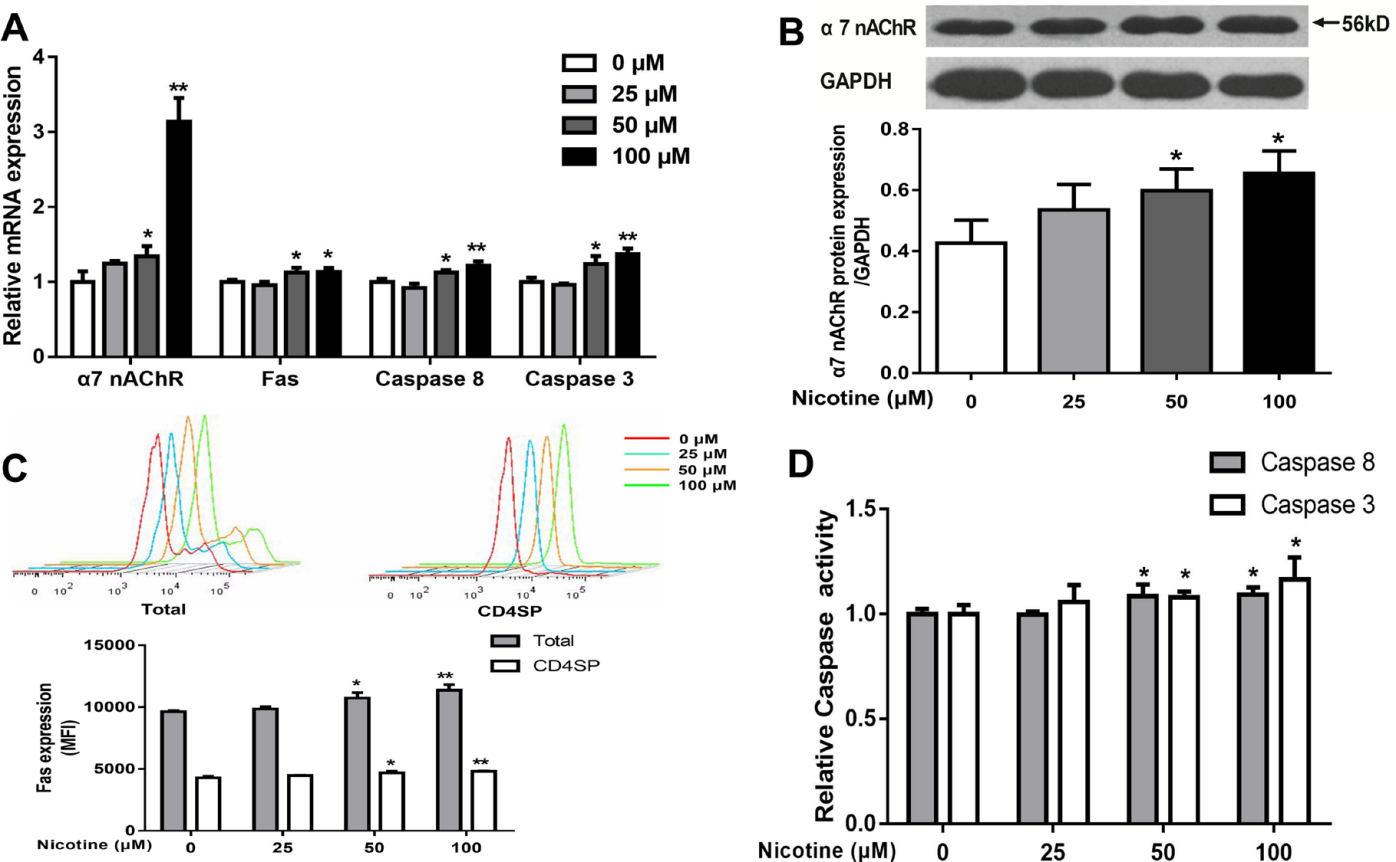
Effects of nicotine (0 to 100 μM) treatment for 48 h on expression of α7 nAChR and expression of Fas apoptotic pathway in primary thymocytes (**A**) mRNA expression of α7 nAChR, Fas, Caspase-8 and Caspase-3 was detected using qPCR in thymocytes. (**B**) a7 nAChR protein expression was determined by western blot in thymocytes. (**C**) Fas protein expression in thymocytes. Thymocytes were stained with CD95-PE-cy7, and the expression of Fas protein was analyzed by flow cytometry. (**D**) The Caspase-8 and Caspase-3 protein activities were detected using the Caspase activity kits. The difference was analyzed with one-way ANOVA. Mean ± SD, *n* = 3–4. ^*^P < 0.05, ^**^P < 0.01 vs control.

The Fas apoptotic pathway was reported to mediate the apoptosis of thymocytes [8], and nicotine exerts its activity by binding α7 nAChR. We then detected the expression of α7 nAChR and Fas and activities of caspase-3 and caspase-8. As shown in Figure 5A, 50 and 100 μM nicotine treatments increased the mRNA expression of α7 nAChR, Fas, casepase-3, and caspase-8, as compared with the control (*P* < 0.01, *P* < 0.05). The protein expression of α7 nAChR and Fas and the activities of caspase-3 and caspase-8 in both the 50 and 100 μM nicotine-treated groups were markedly higher than that of the control (*P <* 0.01, *P <* 0.05, Figure 5B–5D).

#### Nicotine induced CpG demethylation of the Fas promoter

Fas expression is activated by DNA demethylation at transcription level [22]. Therefore, we analyzed the DNA methylation of Fas promoter using MassArray. According to the concentration-dependent results of nicotine on the Fas apoptotic pathway, 50 μM nicotine was selected to treat thymocytes. As shown in Table 1 although the average methylation rates of the Fas promoter were not changed after nicotine treatment, among those CpG sites, nt +295, −2394, and −2441 showed significantly decreased frequency of single CpG methylation after nicotine treatment (*P* < 0.05), and the methylation level on nt +456 also showed a decreasing trend (*P* = 0.06).

**Table 1:** CpG methylation levels in Fas promoter

CpG site (bp)	Control	Nicotine	Nicotine+TET2 siRNA	Nicotine+αBTX
−2469	25.67 ± 0.47	26.33 ± 0.94	25.67 ± 1.25	18.67 ± 8.34
−2441	13.00 ± 0.00	11.00 ± 0.00*	13.00 ± 1.63	10.33 ± 1.25
−2394	10.00 ± 1.63	4.00 ± 0.82*	7.67 ± 3.30	8.00 ± 4.00
−2384	8.00 ± 0.00	8.00 ± 0.82	7.00 ± 1.64	10.50 ± 0.50
−2353	25.67 ± 0.47	26.33 ± 0.94	25.67 ± 1.25	18.67 ± 8.34
−2332	11.00 ± 2.17	13.67 ± 4.64	15.00 ± 3.74	18.00 ± 4.24
−2291	19.67 ± 1.70	18.33 ± 0.47	19.67 ± 0.47	22.00 ± 2.16
−2257:−2261	32.67 ± 2.87	33.67 ± 2.62	30.00 ± 2.94	33.00 ± 1.00
−2164	19.67 ± 1.70	18.33 ± 0.47	19.67 ± 0.47	22.00 ± 2.16
+174	N.D.	N.D.	N.D.	0.67 ± 0.94
+210	8.33 ± 0.94	8.67 ± 0.94	11.00 ± 0.82	9.00 ± 1.41
+250	N.D.	1.33 ± 1.89	N.D.	N.D.
+295	6.00 ± 0.00	4.00 ± 0.00*	8.67 ± 2.05	6.00 ± 2.94
+332	7.67 ± 2.05	7.00 ± 0.82	6.67 ± 0.82	9.00 ± 1.63
+417:+422	4.00 ± 0.82	4.00 ± 1.63	4.00 ± 0.82	3.67 ± 0.94
+456	9.67 ± 1.25	7.00 ± 0.82 ^*p*^ ^= 0.06^	12.00 ± 2.16	12.33 ± 1.89
+464	N.D.	N.D.	N.D.	N.D.
+540	7.00 ± 1.41	6.33 ± 4.11	8.67 ± 0.47	8.33 ± 0.47
+570	N.D.	N.D.	N.D.	N.D.
+596	21.33 ± 2.87	18.67 ± 2.36	18.00 ± 2.45	18.33 ± 2.05
Average	11.47 ± 2.05	10.83 ± 2.09	11.62 ± 1.95	11.43 ± 1.91

The CpG methylation levels in the Fas promoter (−2480 bp to −2141 bp and 174 bp to 596 bp) were detected by MassARRAY method. DATA were analyzed with one-way ANOVA. Mean ± SD, *n* = 3 cell samples per group, *p < 0.05 *vs*. control. N.D.: non-detected.

#### Increased TET2 mediated nicotine-induced Fas promoter demethylation in thymocytes

**Figure 6:**
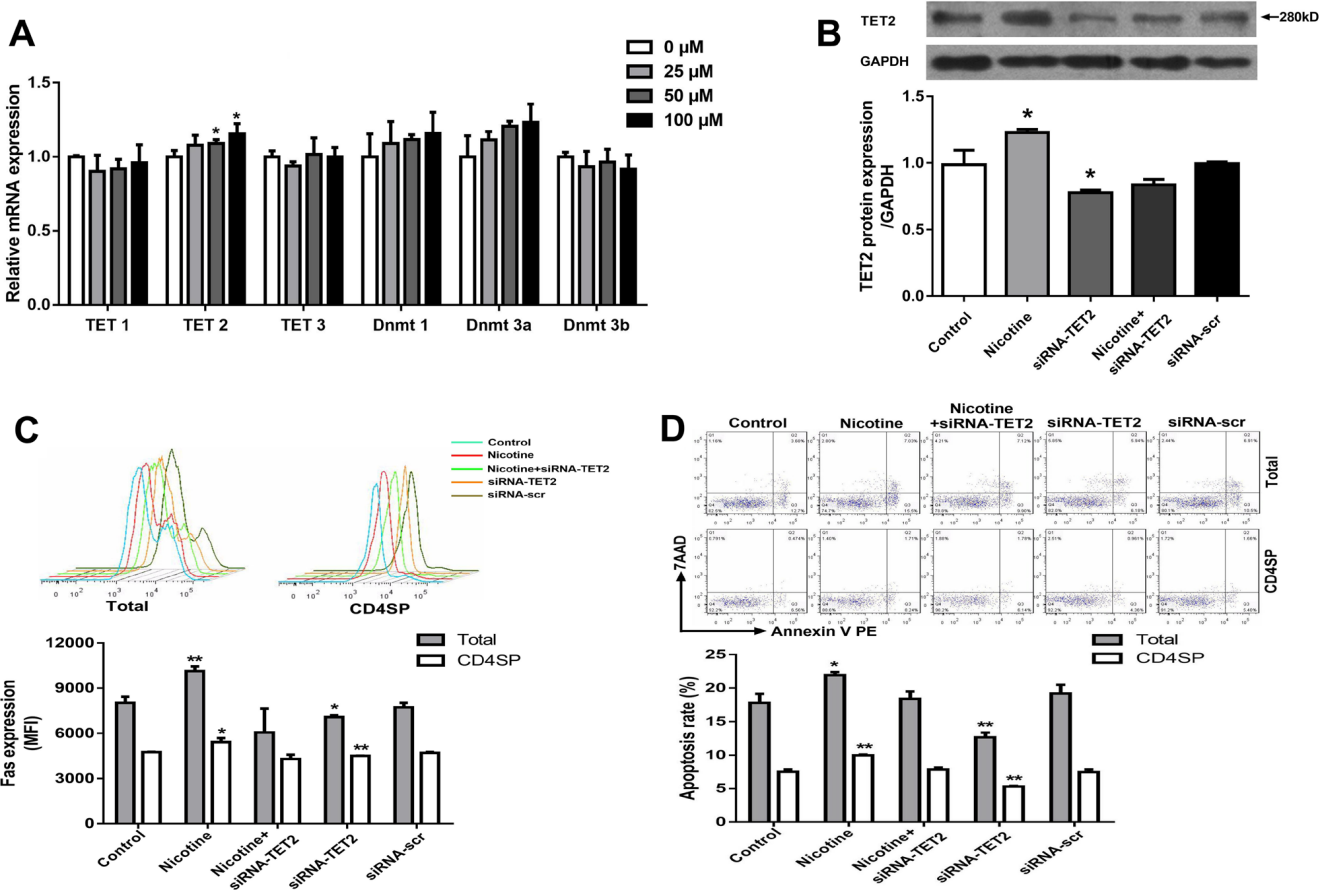
Effects of 50 μM nicotine treatment for 48 hours with or without siRNA-TET2 (100 nM) transfection on thymocyte apoptosis and expression of TET2, α7 nAChR and Fas in primary thymocytes (**A**) The mRNA expression of TET1, TET2, TET3, Dnmt1, Dnmt2a, and Dnmt3b was detected using qPCR. To explore the role of TET2 in the DNA methylation of the Fas gene, TET2 siRNA (100 nM) transfection was performed using Lipofectamine 3000 reagent, and TET2 protein expression was determined by western blot. (**B**) TET2 protein expression. (**C**) Fas protein expression. (**D**) Apoptosis frequency of thymocyte. The difference was analyzed with one-way ANOVA. Mean ± SD, *n* = 3–4. ^*^P < 0.05, ^**^P < 0.01 vs control.

To investigate the mechanisms of nicotine-induced Fas demethylation, the expression of DNA methyltransferases (Dnmts) and TETs, two known key regulators of DNA methylation modification enzymes, were detected. As shown in Figure 6A, the expression of Dnmt1, Dnmt3a, Dnmt3b, TET1, and TET3 in thymocytes showed no difference between the nicotine group and control, but notably increased TET2 expression was observed after 50 μM and 100 μM nicotine treatment (*P* < 0.05). Consistent with this result, an increased TET2 protein level was also observed in nicotine-treated thymocytes (*P* < 0.05, Figure 6B).

To further explore the role of TET2 in nicotine-induced Fas demethylation, thymocytes were transfected by exogenous TET2 siRNA to inhibit TET2 protein expression prior to nicotine treatment. The results showed that the TET2 siRNA transfection clearly reversed the effect of nicotine-promoted TET2 increase and further abolished the single CpG demethylation of nt +295, +456, −2394, and −2441 in the Fas promoter (Table 1). As shown in Figure 6C, TET2 siRNA transfection could also abrogate the nicotine-induced Fas increase. Moreover, data from flow cytometry showed that the TET2 siRNA transfection could also abolish the nicotine-induced thymocyte apoptosis in both total and CD4^+^SP thymocytes (Figure 6D).

#### α7 nAChR mediated nicotine-induced DNA demethylation of the Fas promoter and the following upregulation of Fas-mediated thymocyte apoptosis

**Figure 7:**
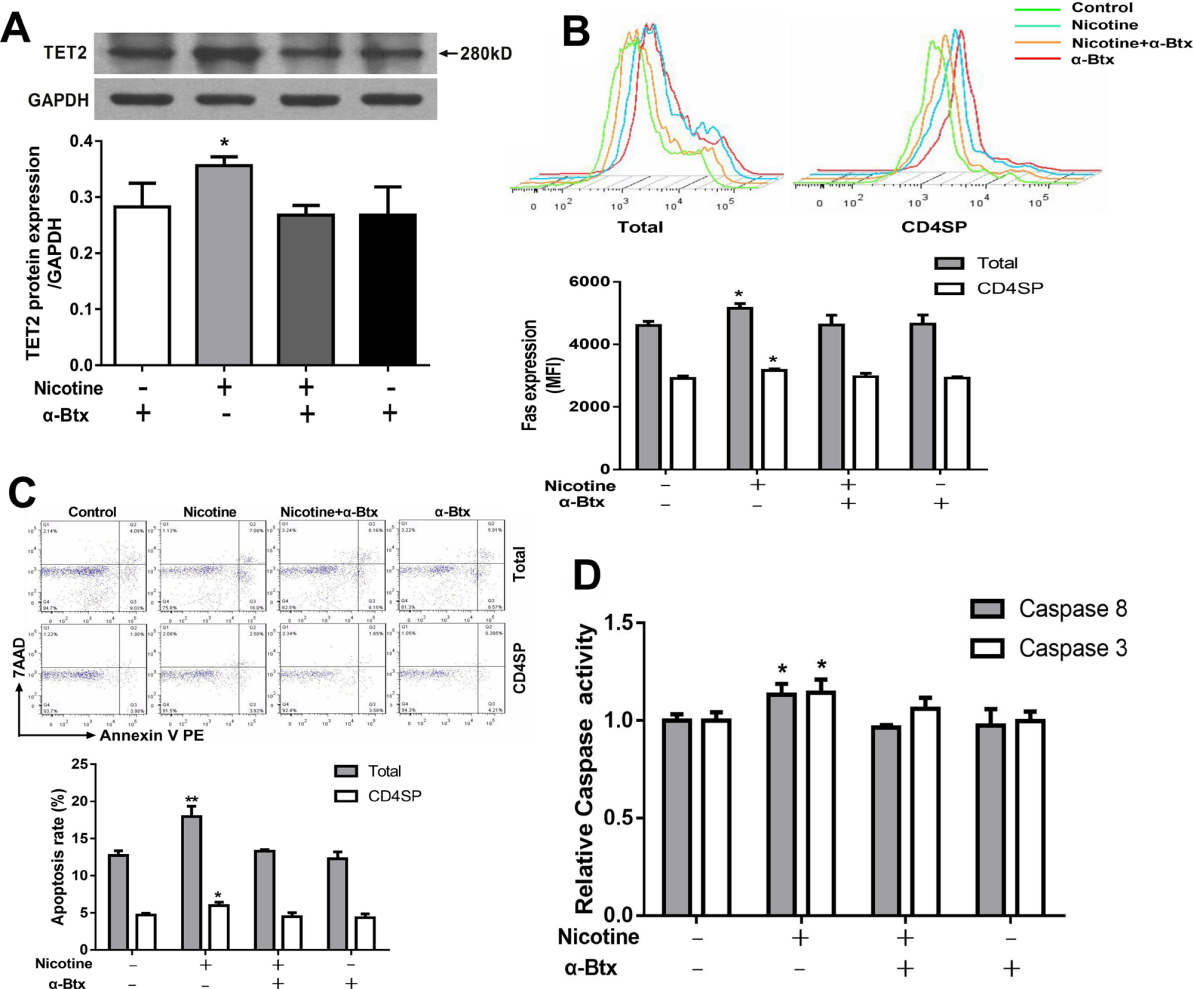
Effects of 50 μM nicotine treatment with or without α-bungarotoxin (α-Btx, 1μg) pretreatment on thymocyte apoptosis and expression of TET2 and Fas apoptotic pathway in primary thymocytes To investigate the role of α7 nAChR in nicotine-mediated apoptosis, thymocytes were pretreated with 1 μg/ml of α-Btx half an hour before nicotine treatment. (**A**) TET2 protein expression was determined by western blot. (**B**) Fas protein expression. (**C**) Apoptosis frequency of thymocyte. (**D**) The Caspase-8 and Caspase-3 protein activities were detected using the Caspase activity kits. The difference was analyzed with one-way ANOVA. Mean ± SD, *n* = 3–4. ^*^P < 0.05, ^**^P < 0.01 vs control.

To further investigate the role of α7 nAChR in TET2-mediated Fas demethylation, thymocytes were pretreated with an α7 nAChR specific antagonist α-bungarotoxin 30 min prior to nicotine treatment. Then TET2 expression, the methylation status of Fas promoter, the expression of the Fas apoptotic pathway and thymocyte apoptosis were determined. As shown in Figure 7A, the expression of TET2 was significantly decreased after α-bungarotoxin pre-incubation compared with the nicotine group (*P* < 0.05). The TET2-induced single CpG demethylation on the nt +295, +456, -2394, and -2441 in the Fas promoter were obviously abolished by pre-incubation of α-bungarotoxin (Table 1). The results of flow cytometry and qPCR further showed that the pre-incubation of α-bungarotoxin clearly abolished the effect of nicotine-induced total and CD4^+^SP thymocyte apoptosis and upregulated Fas, caspase-8 and caspase-3 activities (Figure 7B–7D).

## DISCUSSION

The dose of 3 mg/kg/d of nicotine and the Balb/C strain were widely used to establish the PNE model for representing prenatal cigarette smoke exposure [30–32]. The 3 mg/kg/d dose of nicotine used in this study could equate to a moderate smoker during pregnancy (exposure to 6–8 cigarettes per day) [33, 34]. A mouse embryo starts implantation on GD 4.5 till GD 6.5, and the fetal thymus organogenesis is initiated on GD 11.5 and continues until birth [29, 35]. Therefore, the exposure period from GD 9 to GD 18 not only covered the period of fetal thymus organogenesis and development but also protected the embryo implantation from the influence of nicotine. In fact, no miscarriage or stillborn fetuses have been observed in our study. Nicotine levels in fetal circulation and fetal tissues can be much higher than that in the maternal serum due to free penetration of placenta and lipophilic accumulation of nicotine over the course of 10-month pregnancy, and low enzymatic activity of fetal CYP2A6 [14, 36]. Thus, much higher concentrations up to 100 or even 300 μM have been utilized in many short-term (several days) *in vitro* cell culture studies [37–40]. Therefore, 0–100 μM nicotine treatment were used for mechanism research *in vitro*. The *S*. *pneumoniae* used in the present study is a T cell-dependent antigen, which can induce the immune system to produce cytokines (such as IL-4) and IgG antibodies [34]. It was evident that females are more susceptible to immune disorders. Hence, in this study, female offspring were retained after birth to study the postnatal immunotoxicity of PNE.

Prenatal cigarette smoke exposure could induce thymus atrophy in the fetus and result in allergy-prone deviation in the T-cell response, which manifests as increased IL-4 production during allergic disease development, in postnatal life [28, 41, 42]. Evidence also showed that PNE is related to increased production of inflammatory cytokines after birth [43]. Additionally, Abbas and Vadesilho reported that the increased IL-4 production could cause an abnormal increase of the ratio of IgG1/IgG2, which in turn polarized the offspring immune system toward immune diseases [44, 45]. Additionally, our previous study showed that the PNE male offspring showed an increased IgG1/IgG2a ratio and higher IL-4 content after antigen stimulation [34]. In this study, the changes in the IgG1, IgG2a, and IL-4 contents in PNE female offspring were different from those of the males. After *S. pneumoniae* immunization, the female PNE offspring also showed increased IL-4 content and an increased ratio of IgG1/IgG2. These findings confirmed our hypothesis that PNE could cause immune disorders in offspring.

Accelerated apoptosis of fetal thymocytes is one of the most important reasons for postnatal immune impairments induced by prenatal adverse factors, such as smoke, glucocorticoids, TCDD and diethylstilbestrol [46–50]. In our study, the apoptosis percentages of fetal total and CD4^+^SP thymocytes in PNE fetus and in PNE offspring were higher than that of control. Moreover, the reduced cell proportion of CD4^+^SP was consistent with the increased apoptosis at the two time points. These findings suggested that PNE could decrease CD4^+^SP through increasing its apoptosis percentages, and this effect could persist in postnatal life. Due to CD4^+^SP thymocyte development is essential for the establishment of the CD4^+^T helper cell immune network [45]. Researchers have found that the reduction of periphery CD4^+^T cells was tightly related to increased IL-4 and IgG1 production following immunization in offspring [51]. This persistent increase of CD4^+^SP apoptosis in our study might be the potential explanation for PNE-induced immune impairments in female offspring.

In recent years, different studies have presented conflicting data while investigating the effects of nicotine on cell apoptosis. These contradictory results may be due to the different duration of exposure and the maturation of cells [15]. In *in vivo* studies, almost all animal models that had a long exposure time or high dose of nicotine showed pro-apoptotic effects [15]. Additionally, researchers reported that nicotine showed a more typical anti-apoptotic effect on well-developed cells but a strong pro-apoptotic effect on immature or developing cells [19, 52, 53]. In *in vitro* studies, Middlebrook et al. reported that the pro-apoptotic effects of nicotine might act only on immature thymocytes or thymocyte precursors [16]. The increasing doses (10^–8^–10^–4^ M) of nicotine showed different effects on cell validity and the lower dose of nicotine treatment exerts higher anti-apoptotic effects on thymoma cells [54]. Additionally, the thymopoiesis process is tightly controlled, and almost 95% of thymocytes were eliminated through apoptosis during this process [29]. Thymocytes are vulnerable to xenobiotics during the thymopoiesis [1]. In the present study, the thymocyte is the immature immune cell, and nicotine is easier to accumulate and more difficult to be remove in fetus [14, 36]. Nicotine exposure from GD 9 to GD 18 may provide a “long-term exposure to a high dose of nicotine” micro-environment for thymocytes and lead to apoptosis. The results from our *in vitro* data further confirmed that exposure to nicotine for a longer duration or at a higher dose could induce more significant apoptosis of both total and CD4^+^SP thymocytes.

In the immune system, nicotine induces its actions by binding to α7 nAChR, which is involved in directly inducing the apoptosis of immune cells [15]. Slotkin *et al.* showed that prenatal nicotine-induced apoptosis was mediated *via* nAChR activation and increased nAChR concentrations [55]. In addition, Vivek *et al.* also reported that the expression of α7 nAChR was increased following prenatal cigarette exposure and it coincided with the changes of caspase expression [19]. Consistent with these studies, after 0–100 mM nicotine treatments *in vitro* in the present study, the mRNA and protein expression of α7 nAChR were significantly increased in a concentration-dependent manner and were associated with upregulated Fas, caspase-3 and caspase-8 in thymocytes. Further, the α7 nAChR antagonist α-bungarotoxin not only abrogated nicotine-mediated pro-apoptotic effects but also reversed the nicotine-upregulated Fas apoptotic pathway. These findings suggested that α7 nAChR might be indispensable in Fas-mediated pro-apoptotic effects in nicotine-treated thymocytes.

Our previous studies showed that PNE could regulate DNA methylation of genes in developing organs (such as StAR and acetoacetyl-CoA in fetal adrenal) [25, 26]. DNA methylation is a major epigenetic mechanism that control Fas gene expression at the level of transcription [22, 56]. And both Gazin and Thaler reported that two regions in the Fas promoter (−2,600 bp to −2200 bp; −30 bp to 623 bp) are rich in CpG sites [56, 57]. Our pre-experiment further confirmed that the CpG dinucleotide centered on this two regions (−2480 bp to −2141 bp and 174 bp to 596 bp) in the Fas promoter (Supplementary Figure 1). Accordingly, the methylation status within these two CpG-rich regions was detected in the present study, and the results showed that the CpG methylation levels on nt +295, −2394, and −2441 were significantly decreased, which indicated that nicotine could induce Fas expression through promoting DNA demethylation of the Fas promoter.

Two families (Dnmt family enzymes; TET family enzymes) of enzymes have been identified as regulating DNA methylation and demethylation [58]. In the present study, we first detected mRNA expression of Dnmts and TETs to preliminarily identify which enzyme might be the mediator of the methylation modification of the Fas promoter in thymocytes following nicotine treatment. We found that only TET2 expression increased significantly. The western blot analysis also showed a dose-dependent increase in protein expression of TET2 after nicotine treatment. Moreover, the results of a TET2 siRNA transfection experiment showed abolished nicotine-induced Fas demethylation, which further confirmed that TET2 mediated the nicotine-induced DNA demethylation of the Fas promoter. Now there is lacking of knowledge about how TET2 demethylates FAS yet. During T cell development, two key transcription factors, Smad3 and STAT5, were reported to be involved in promoting the TET2 demethylation [59]. Following TET increases, Smad3 could facilitate the TET1 and TET2 binding to the promoter of target genes [59]. Another study also showed that the Smad3 was an upstream signal of the Fas apoptotic pathway, and the Smad3 knockdown could effectively inhibited the Fas expression and the caspase activation [60]. Thus, we supposed that, in thymocytes, the increased TET2 might also be facilitated by Smad3 onto Fas promoter and then induce Fas promoter demethylation. Although no report is available on whether α7 nAChR activation is associated with TET2 expression, Nair VS *et al.* reported that TCR signaling could upregulate TET2 expression during T cell development [61]. It was reported that α7 nAChR could release similar signals in thymocyte as TCR signaling [16, 62]. As a TCR downstream signaling, NF-κB signaling was reported to be widely involve in thymocyte development following the TCR activation [63, 64]. A study published recently showed that TET2 expression could be induced by LPS stimulation in an NF-κB-dependent manner [65]. Additionally, it was proved that the α7 nAChR activation could upregulate the NF-κB signaling [66–68]. Hence, the NF-κB signaling might be the downstream of α7 nAChR to activate TET2. In the present study, we found that α-bungarotoxin clearly abolished the effect of nicotine-promoted TET2 expression, which confirmed that TET2 expression was activated by α7 nAChR. Further, with the pre-incubation of α-bungarotoxin, the methylation level of Fas in the nicotine group is comparable with the control. These findings suggested that α7 nAChR mediated nicotine-induced TET2 expression and the following Fas promoter demethylation.

**Figure 8:**
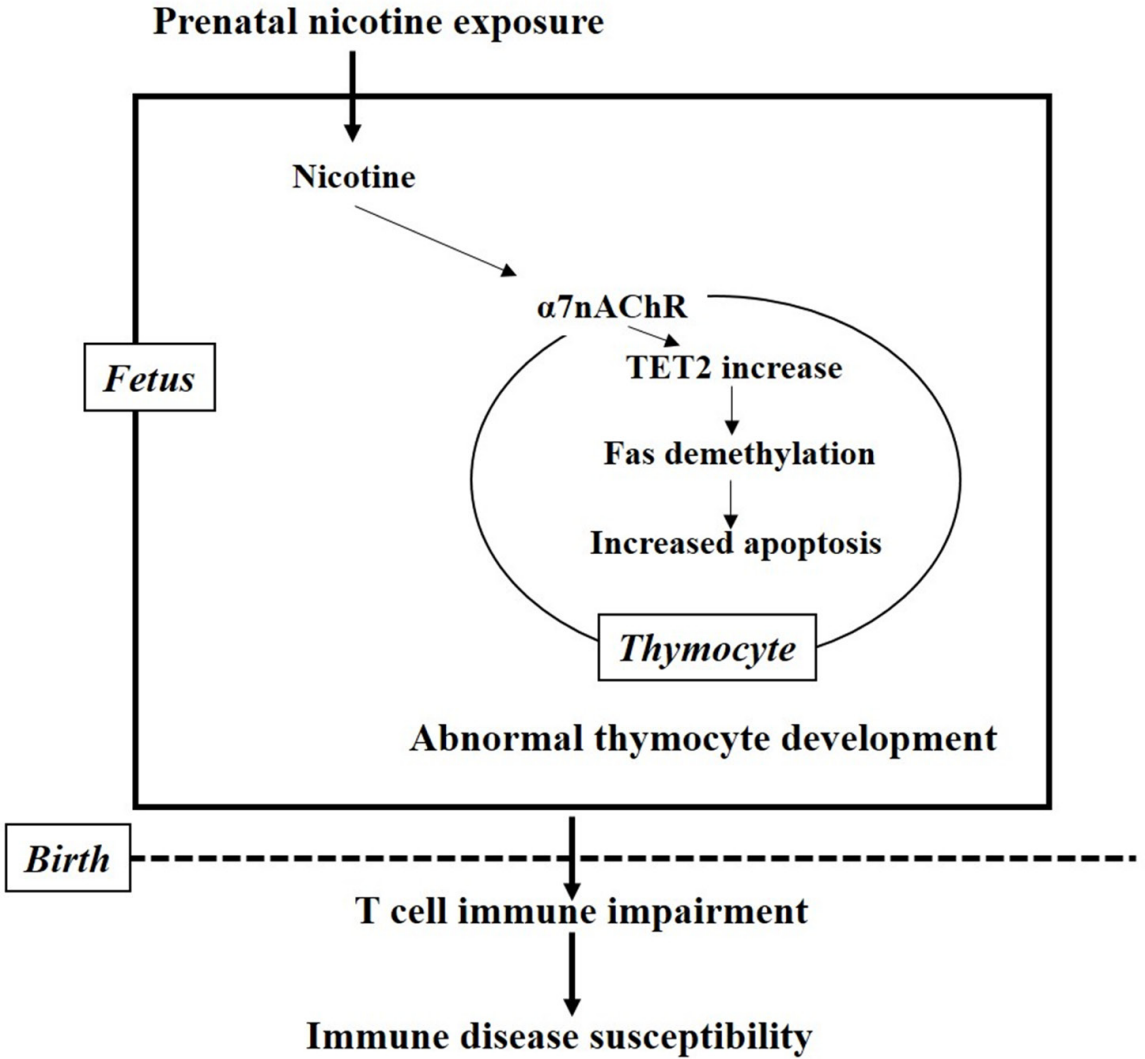
The mechanism hypothesis for the present study Nicotine activates the α7 nAChR on thymocyte and increases the expression of TET2, which in turn induce the demethylation of the Fas promoter followed by increased Fas expression and upregulated Fas apoptotic pathway in thymocyte. The increased fetal thymocyte apoptosis finally results in immune impairment and susceptibility to immune diseases after birth.

In summary, PNE directly increased the apoptosis of total and CD4^+^SP thymocyte in the fetus, which persisted into postnatal life and resulted in immune impairments in female offspring. The underlying molecular mechanisms (Figure 8) of the pro-apoptotic effects of nicotine might be that nicotine activates the α7 nAChR on thymocyte and increases TET2 expression, which could subsequently induce DNA demethylation of the Fas promoter followed by increased Fas expression and an upregulated Fas apoptotic pathway in thymocyte. Our findings showed, for the first time, that α7 nAChR could increase TET2 expression and Fas demethylation, and provided an epigenetic mechanism for elucidating the pro-apoptotic effects of nicotine on thymocytes. Our results also provided evidence for exploring the developmental origin of immune disease susceptibility in PNE offspring.

## MATERIALS AND METHODS

### Chemicals and reagents

Nicotine and α-bungarotoxin were purchased from Sigma-Aldrich (St. Louis, MO, USA). Monoclonal antibodies (anti-mouse CD3-FITC, anti-mouse CD4-APC, anti-mouse CD8-PE-cy7, Rat IgG2b K Isotype Control FITC, Rat IgG2b K Isotype Control APC and Rat IgG2a K Isotype Control PE-cy7) and Annexin V/PE Apoptosis Detection kit were purchased from eBioscience (San Diego, USA). Anti-mouse CD95-PE-cy7 was obtained from BD Biosciences (New Jersey, USA). Mouse IgG1 and IgG2a ELISA kits were obtained from MultiSciences (Hangzhou, Zhejiang, China). Mouse IL-4 ELISA kit was obtained from Dakewe Biotech (Shenzhen, Guangdong, China). Anti-a7 nAChR and anti-TET2 were obtained from Abcam (San Diego, USA). Trizol was purchased from Life Technologies (Gaithersburg, MD, USA). Reverse transcription and RT-qPCR kits were purchased from TaKaRa Biotechnology (Dalian, Liaoning, China). RPMI-1640 medium and fetal bovine serum (FBS) were purchased from HyClone (Logan, UT, USA). Caspase-3 Action Detection kit and Caspase-8 Action Detection kit were from Beyotime Biotechnology (Shanghai, China). The TET2 siRNA and all primers were synthesized by Sangon Biotech Co., Ltd. (Shanghai, China). The TIANamp Genomic DNA Kit was from TIANGEN Biotech Co., Ltd. (Beijing, China). All chemicals and reagents were analytical grade.

### Animals

The animal experiments were approved by the Committee on the Ethics of Animal Experiments at the Wuhan University School of Medicine (Permit Number: 14016). All protocols were performed according to the Guidelines for the Care and Use of Laboratory Animals of the Chinese Animal Welfare Committee and the International Council on Research Animal Care. The virgin female (22 ± 2 g) and male (25 ± 2 g) Balb/C mice for *in vivo* experiment and the infant mice at age of 3 weeks (14 ± 1g) for *in vitro* experiment were obtained from the Experimental Center of Hubei Medical Scientific Academy (No. 2008–0005, Wuhan, Hubei, China). The animal experiments were performed in the Center for Animal Experiment of Wuhan University (Wuhan, Hubei, China), which has been accredited by the Association for Assessment and Accreditation of Laboratory Animal Care International (AAALAC).

### *In vivo* experiments

#### Animal treatment

As shown in Supplementary Figure 2, female mice were mated with male mice at 2:1 overnight, after a one-week acclimation. The day was declared as GD 0 by the presence of a vaginal plug in the vagina. The pregnant mice were randomly assigned to the PNE group or control group. The pregnant mice of the PNE group were subcutaneously administered 1.5 mg/kg of nicotine twice per day from GD 9 to GD 18. The mice of the control group were administered the same volume of saline at the same frequency and intervals. On GD 18, some pregnant mice were sacrificed under isoflurane anesthesia and then the fetuses from 3 litters (each litter 6–8 fetuses) per group were obtained and decapitated after euthanasia. The fetal thymus from each littermate was collected and pooled into one sample. These 3 pooled thymus samples per group were used for flow cytometry detection as a three-time repeated experiment.

The other pregnant mice were housed until delivery, and on PND 0, the numbers of infants were normalized to 6 infants per dam to assure adequate and standardized nutrition. The female offspring were weaned on PND 21. On PND 42, half of the female offspring (8–10 mice per group) were subcutaneously administered an *S*. pneumoniae vaccine to establish an inflammation model. Then, the animals were anesthetized with isoflurane and sacrificed to collect blood samples 7 days later (on PND 49). The thymuses of 3 female offspring per group were harvested for flow cytometry. Serum (from 8–10 female offspring per group) was stored at −80°C for measurement of IL-4, IgG1, and IgG2a concentrations.

#### Flow cytometry

The thymus was cut into small pieces and gently mashed through a 40 μm stainless steel sieve. Single cells were obtained by centrifugation at 335 × g for 5 min at 4°C and resuspended at 1 × 10^7^ cells/ml in flow cytometry staining (FCS) buffer (PBS, 1% FBS, 0.02% sodium azide). A cell apoptosis assay was performed using flow cytometry according to the method previously described [34]. Briefly, thymocytes were stained with 100 μl of antibody cocktail (FITC-CD3, APC-CD4 and PE-cy7-CD8) in FCS buffer for 30 min on ice in the dark. After surface staining, the cells were washed and incubated with 100 μl of fluorochrome-conjugated Annexin V at room temperature for 10 min. Then, the cells were washed and resuspended in 200 μl of binding buffer. 7-Aminoactinomycin D (7-AAD) was added before being analyzed by flow cytometry. Samples were determined with a BD FCS AriaTM III flow cytometer (BD Biosciences). Flow cytometry data were analyzed and plotted using FlowJo software. For Fas analysis, CD95 expression was also detected.

#### Measurement of total IgG1, IgG2a antibodies and IL4 in serum on PND49

The levels of IL-4, total IgG1 and total IgG2a in offspring serum were determined using ELISA kits according to the manufacturer’s protocols. The dilution ratio of serum samples for IgG1 detection was 1:10000, and for IgG2a was 1:2000. There was no dilution for IL-4 detection.

### *In vitro* experiments

#### Thymic primary cell culturing and treatment

Thymocyte development reaches its peak during adolescence (in mice, 5 weeks), and then increased sex hormones lead to physiological thymus involution and massive thymocyte apoptosis [29]. Therefore, to obtain thymocytes in the best condition and to avoid excessive physiological apoptosis, we obtained primary thymocytes from mice at 3 to 4 weeks to explore the direct effect and mechanisms of nicotine on thymocyte apoptosis. Thymus lobes dissected from infant mice at age 3 weeks were placed on a stainless steel sieve in a 6-well plate in 1 ml of complete RPMI1640 medium (supplemented with 20% FBS) and were gently dissociated over the screen using the plunger of a 2 mL syringe. Cells were washed in medium for 5 min, 500 × g at 4°C and cultured in complete medium at 37°C in 5% CO_2_. Cells were synchronized by serum starvation for at least 12 h before treatment with nicotine for the indicated periods or concentrations. The sample sizes of cells per group were 3–4 for all *in vitro* experiments. To study the dose-effect and time-effect of nicotine on thymocyte apoptosis, the cells were treated with the different concentrations (25, 50 and 100 μM) of nicotine for 48 h or 50 μM of nicotine for 24 h, 48 h and 72 h (Supplementary Figure 2). To investigate the role of α7 nAChR in nicotine-mediated apoptosis, the cells were pretreated with 1 μg/ml of α-bungarotoxin, an α7 nAChR specific antagonist, 30 min before nicotine treatment.

#### siRNA-TET2 transfection

To explore the role of TET2 in the DNA methylation of the Fas promoter, TET2 siRNA transfection using Lipofectamine 3000 reagent was performed according to the manufacturer’s protocol. Briefly, cells were seeded on 96-well plates at a density of 5 × 10^6^/ml and cultured for 24 h before transfection. The siRNA and Lipofectamine 3000 were diluted in Opti-MEM medium without serum, then the dilution of siRNA and Lipofectamine 3000 were mixed at a ratio of 1:3 w/v. This mixture was stored at room temperature for 15 min and added to 96-well plate by dispensing 100 nM siRNA per well. Cells were harvested for analysis 24 h after transfection.

#### Flow cytometry

For *in vitro* experiments, the cells were also rinsed with FCS buffer and resuspended at 1 × 10^7^ cells/ml in FCS buffer. A cell apoptosis assay was performed as described above. For Fas analysis, CD95 expression was also detected.

#### Caspase-3 and caspase-8 activity assay

The activities of caspase-3 and caspase-8 were determined using the Caspase Action kits according to the manufacturer’s protocols. Assays were performed on 96-well plates by incubating 10 μl thymocyte lysate per sample in 80 μl reaction buffer (1% NP-40, 20 mM Tris–HCl (pH 7.5), 137 mM Nad and 10% glycerol) containing 10 μl substrate (Ac-DEVD-pNA) (2 mM). The lysates were incubated at 37°C for 4 h and measured with an ELISA reader at an absorbance of 405 nm.

#### Western blot

Proteins were obtained by lysis buffer as previously described [69]. Proteins were loaded onto SDS-PAGE gels for electrophoresis and then transferred onto PVDF membranes. After blocking in 5% fat-free milk in TBST for 1.5 h, the membranes were incubated with primary antibody (anti-a7 nAChR, anti-TET2) at 4°C overnight. Subsequently, the membranes were incubated with corresponding HRP-conjugated secondary antibodies at room temperature for 1.5 h. After washing 3 times with TBST (for 10 min each), bound antibodies were visualized using enhanced chemiluminescence. Glyceraldehyde phosphate dehydrogenase (GAPDH) was used as a loading control.

#### RNA preparation and qPCR

Total RNA was isolated from the thymocytes using Trizol reagent according to the manufacturer’s protocol. The concentration and purity of the isolated RNA were determined by a spectrophotometer, and the concentration of each sample was adjusted to 1 μg/μl. Single-strand cDNA was prepared using a reverse transcription kit. All primer sequences shown in Supplementary Table 1 were designed by Primer Premier 5.0 from PREMIER Biosoft International (Palo Alto, CA, USA) and queried by NCBI BLAST database for homology comparison. PCR assays were performed using a QuantStudio 6 Flex from Applied Biosystems (Foster City, CA, USA) in a total volume of 20 μl reaction mixture containing 1 μl of cDNA template, 0.4 μl of 10 μM each primer, 10 μl of 2 × Premix Ex Taq, 0.4 μl of ROX and 7.8 μl of DEPC-H_2_O. PCR cycling conditions were as follows: 30 s at 95°C for pre-denaturation, 5 s at 95°C for denaturation, and appropriate annealing conditions for each gene (Supplementary Table 1). The ranges of the CT values of GAPDH were 20–22. The ranges of the Ct values of the other genes were 26–31. The mRNA expression levels were calculated using the ΔΔCt method using GAPDH as an internal control. The relative mRNA expression levels were standardized by setting the corresponding gene expression of the control group as 1.

#### Genomic DNA extraction and DNA methylation assay based on MassARRAY

Genomic DNA was isolated from the thymocytes using the TIANamp Genomic DNA Kit, according to the manufacturer’s protocol. The quality and quantity were evaluated by a spectrophotometer. Isolated Genomic DNA samples were bisulfite-treated to convert all C bases that are not methylated to U bases. PCR primers for the Fas gene were designed using Sequenom’s EpiDesigner (Sequenom). Two primers were used for PCR amplification (Fas1 and Fas2), which cover the CpG-rich region in the Fas promoter. Primers for Fas1 (-2480 bp ∼ -2141 bp) were as follows: forward: 5′- aggaagagagAGTGTGGTTGGTATTGGGTGTTAT-3′; reverse: 5′-cagtaatacgactcactatag ggagaaggctAATTCAACACATCCACAATTTAACAA-3′. Primers for Fas2 (174 bp ∼ 596 bp) were as follows: forward: 5′-aggaagagagAAAAGGGAAAATTTTTATTTGTTGTG-3′; reverse: 5′- cagtaatacgactcactatagggagaaggctCAATAAATTTTACAAACCTTCTAACCC-3′. The bisulfite-converted DNA was PCR amplified (94°C for 4 min; 45 cycles of 94°C for 20 s, 60°C for 30 s, and 72°C for 1 min; 72°C for 3 min), and then the amplified products were transcribed into RNA fragments using the T7 RNA polymerase (T-cleavage transcription, 37°C for 180 min). Mass spectra were acquired using a MassARRAY MALDI-TOF MS (Sequenom). According to the 16 Da molecular weight differences between CpG and CpA, peak detection and methylation ratio calculations were performed using EpiTyper software (Sequenom).

#### Statistical analysis

The sample sizes (n) of animals per group for IL-4, IgG1, and IgG2a detection were 8–10. For the *in vivo* flow cytometry experiments and all of the *in vitro* experiments, the sample sizes (n) were 3–4 per group. All measurement data were expressed as the mean ± SD. The difference between two groups was compared using the *t*-test, and the difference among multiple groups was analyzed with one-way ANOVA. Values of *P* < 0.05 were considered statistically significant. Data were analyzed using SPSS 17 (SPSS Science Inc., Chicago, Illinois) and Prism (GraphPad Software, Inc., La Jolla, CA, USA, version 5.0).

## SUPPLEMENTARY MATERIALS FIGURES AND TABLE



## Abbreviations

DN: CD4^−^CD8^−^ double negative thymocytes
Dnmt: DNA methyltransferase
DP: CD4^+^CD8^+^ double positive thymocytes
FBS: fetal bovine serum
FCS: flow cytometry staining
GAPDH: glyceraldehyde phosphate dehydrogenase
GD: gestational day
IL: interleukin
Ig: immunoglobulin
nAChR: nicotinic acetylcholine receptor
PBS: phosphate-buffered saline
PNE: prenatal nicotine exposure
PND: postnatal day
RT-PCR: reverse-transcription PCR
SP: single positive thymocytes
*S. pneumoniae*: streptococcus pneumoniae vaccine
TET: tet methylcytosine dioxygenase
