# Supplementary material for: α7 nAChR mediated Fas demethylation contributes to prenatal nicotine exposure-induced programmed thymocyte apoptosis in mice
Source: Oncotarget. 2017 Oct 5;8(55):93741–56. doi: 10.18632/oncotarget.21526 (PMC5706832; doi:10.18632/oncotarget.21526)
Supplement: Supplementary file 1 [file oncotarget-08-93741-s001.pdf]

α7 nAChR mediated Fas demethylation contributes to prenatal nicotine exposure-induced programmed thymocyte apoptosis in mice

SUPPLEMENTARY MATERIALS

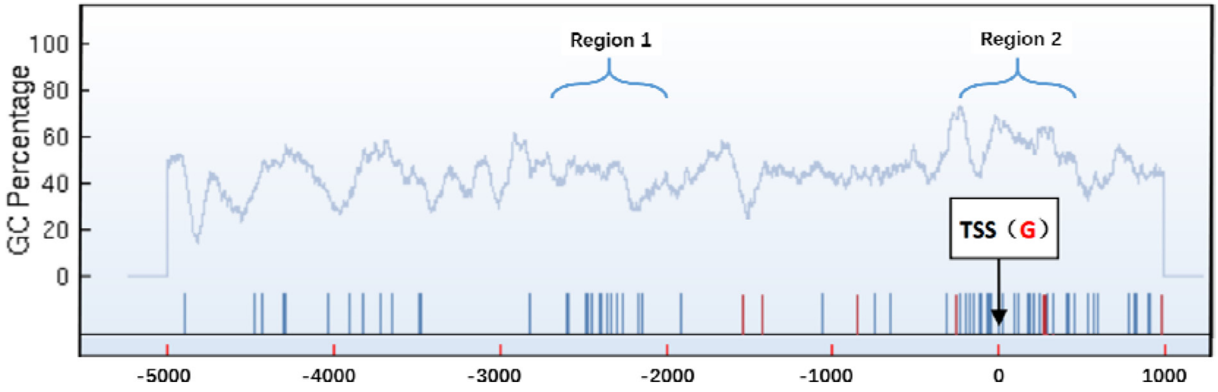

Supplementary Figure 1: Two CpG-rich regions in the Fas promoter. CG dinucleotide positions were shown by vertical lines.

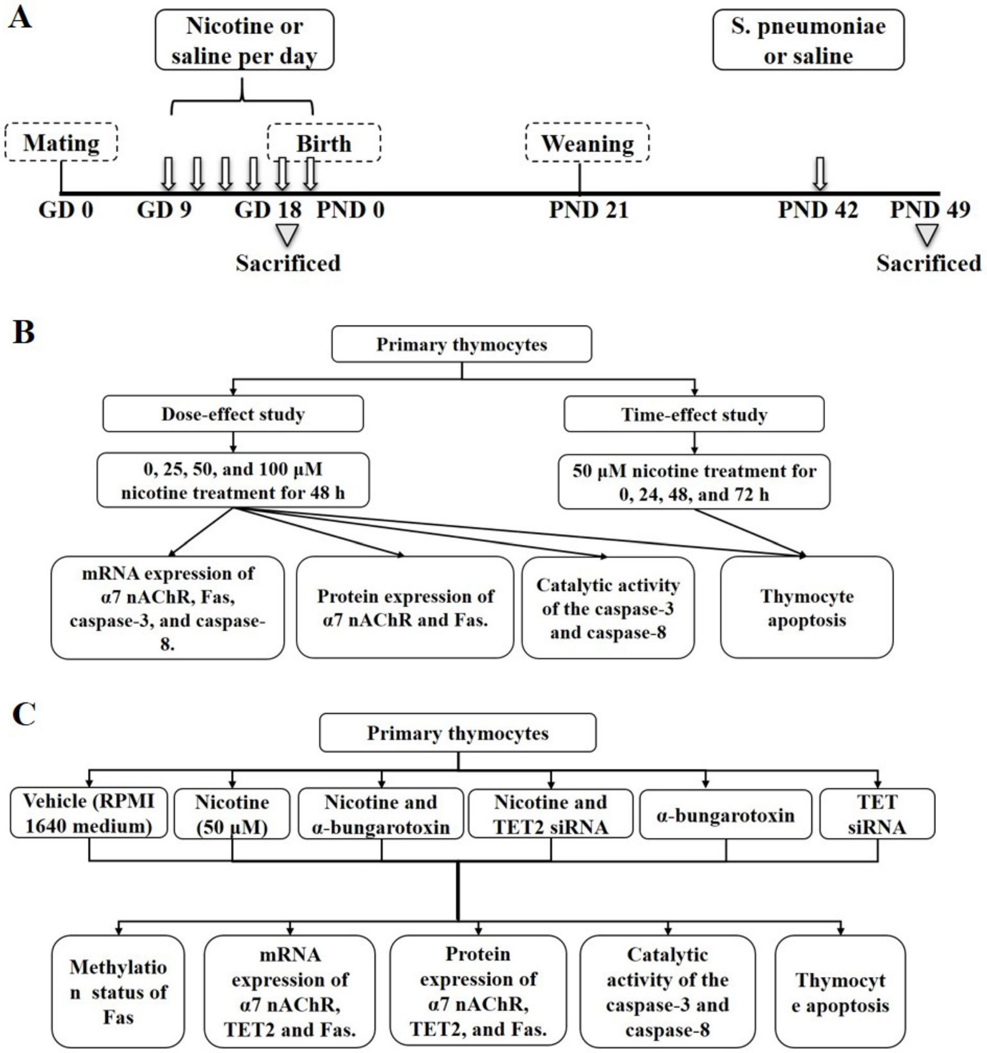

Supplementary Figure 2: The experimental flow diagram. (**A**) Nicotine or saline was administered to pregnant mice from gestational day (GD) 9 to GD 18. On postnatal day (PND) 42, an inflammation model was established via S. pneumoniae immunization. Offspring were sacrificed for test on GD 18 and PND 49; (**B**) The primary thymocytes were treated with different concentrations of nicotine (0, 25, 50, and 100 μM) for 48 h, and treated with 50 μM nicotine for different durations (0, 24, 48, and 72 h) to investigate the dose-effects and the time-effects of nicotine on thymocyte apoptosis, respectively; (**C**) The primary thymocytes were treated respectively with vehicle (RPMI 1640 medium), nicotine, nicotine and α-bungarotoxin, nicotine and TET2 siRNA, α-bungarotoxin, and TET2 siRNA to study the molecular mechanisms of nicotine's pro-apoptotic effects on thymocytes.

Supplementary Table 1: Primers and PCR conditions of mouse used in quantitative real-time PCR

Genes	Forward primer	Reverse primer	Product (bp)	Annealing
Caspase-3	CGTGGTTCATCCAGTCCCTTT	ATTCCGTTGCCACCTTCCT	102	60°C, 30 s
Caspase-8	AGGTACTCGGCCACAGGTTA	TGGGATGTAGTCCAAGCACA	137	60°C, 30 s
Fas	ATGCACACTCTGCGATGAAG	CAGTGTTCACAGCCAGGAGA	120	60°C, 30 s
DNMT1	AAGAATGGTGTTGTCTACCGAC	CATCCAGGTTGCTCCCCTTG	178	60°C, 30 s
DNMT3a	GAGGGAACTGAGACCCCAC	CTGGAAGGTGAGTCTTGGCA	216	62°C, 30 s
DNMT3b	AGCGGGTATGAGGAGTGCAT	GGGAGCATCCTTCGTGTCTG	72	62°C, 30 s
TET1	ACACAGTGGTGCTAATGCAG	AGCATGAACGGGAGAATCGG	112	60°C, 30 s
TET2	AGAGAAGACAATCGAGAAGTCGG	CCTTCCGTACTCCCAAACTCAT	104	62°C, 30 s
TET3	TGCGATTGTGTCGAACAAATAGT	TCCATACCGATCCTCCATGAG	111	62°C, 30 s
GAPDH	AACTTTGGCATTGTGGAAGG	GGATGCAGGGATGATGTTCT	132	60°C, 30 s

DNMT: DNA methyltransferase; TET: Tet methylcytosine dioxygenase; GAPDH: glyceraldehyde phosphate dehydrogenase.
